# Smart Technologies in the Management of Metabolic Syndrome and Oral Health: A Narrative Review

**DOI:** 10.3390/healthcare14213770

**Published:** 2026-11-04

**Authors:** Anna-Maria-Clara Gherbon, Deiana Cuț, Romulus Timar, Oana Albai

**Affiliations:** 1 Doctoral School of Medicine, “Victor Babeș” University of Medicine and Pharmacy, 300041 Timișoara, Romania; 2 Department XVI Rheumatology, “Victor Babeș” University of Medicine and Pharmacy, 300041 Timișoara, Romania; 3 Railway Clinical Hospital, 300173 Timișoara, Romania; 4 Department VII Diabetes, Nutrition, and Metabolic Diseases, “Victor Babeș” University of Medicine and Pharmacy, 300041 Timișoara, Romania; timar.romulus@umft.ro (R.T.); albai.oana@umft.ro (O.A.); 5 Diabetes, Nutrition, and Metabolic Diseases, “Pius Brînzeu” Emergency Hospital, 300723 Timișoara, Romania

**Keywords:** metabolic syndrome, periodontitis, oral health, machine learning, artificial intelligence, wearable electronic devices, telemedicine, saliva, chronic disease management

## Abstract

**Highlights:**

**What are the main findings?**

- Smart technologies developed with metabolic syndrome (MetS) as the main focus are abundant, as are those that focus on oral disease, though few studies combine oral and metabolic data, and those that do are mostly cross-sectional.
- The vast majority of MetS prediction models were validated only internally, and several used the diagnostic components of MetS as predictors.

**What are the implications of the main findings?**

- Dental visits could, in principle, become an additional point of metabolic risk assessment; however, this has not yet been tested prospectively, and salivary markers have not been validated for screening.
- Prospective cohorts, externally validated models and trials of integrated dental–medical care pathways are needed before these approaches can be introduced as routine tools.

**Abstract:**

**Background/Objectives**: Metabolic syndrome (MetS) and periodontitis are two of the most common chronic conditions sharing an underlying mechanism represented by systemic inflammation. Smart technologies, including machine learning (ML), wearable and Internet of Things (IoT) devices, telemedicine and intraoral sensors, are increasingly used in both fields. This narrative review examined how these technologies are used to predict, monitor and manage MetS and oral diseases and what evidence supports combining oral and metabolic data. **Methods**: Following the SANRA framework, PubMed, Scopus and Web of Science were searched for English-language articles published between January 2021 and May 2026. Of the 6922 records identified (3520 after de-duplication), 1852 were screened by title and abstract, and studies were selected by relevance to three research questions. Applications were classified by their current level of evidence. **Results**: ML models for MetS prediction often reported high discrimination (area under the receiver operating characteristic curve [AUC] up to 0.99), but most were validated only internally, and several used the diagnostic components of MetS as predictors. Wearable, telemedicine and mobile interventions, usually combined with lifestyle programs, were associated with improvements in body weight, blood pressure and glycemic markers; artificial intelligence tools for dental imaging showed accuracy comparable to clinicians in mostly retrospective studies, with limited evidence on patient outcomes. Few studies combined oral and metabolic data: cross-sectional ML studies identified periodontal severity as a predictor of MetS and metabolic conditions as predictors of periodontitis, and salivary glucose correlated with blood glucose mainly in people living with diabetes, which does not establish diagnostic accuracy. No prospective study has tested an integrated dental–medical pathway. **Conclusions**: Integrating oral and metabolic data through smart technologies is biologically plausible and appears technically feasible, but its added diagnostic value, clinical effectiveness and cost effectiveness have not been established. Externally validated models, prospective cohorts and trials of integrated care pathways are required before routine use.

## 1. Introduction

Metabolic syndrome (MetS) represents a cluster of cardiometabolic risk factors, including abdominal obesity, dyslipidemia, hypertension and insulin resistance, that increases the risk of developing type 2 diabetes and cardiovascular disease. Its prevalence is rising worldwide, and chronic systemic inflammation is considered a central mechanism [1,2]. In this review, MetS is understood according to the harmonized criteria, under which the syndrome is present when at least three of five components are met: elevated waist circumference (using population-specific cut-offs), elevated triglycerides, reduced high-density lipoprotein (HDL) cholesterol, elevated blood pressure and elevated fasting glucose [3]. The studies discussed below applied different definitions, including the NCEP ATP III and IDF criteria, which limits direct comparison of their results.

Periodontal disease and MetS are linked in both directions: local periodontal inflammation may worsen systemic metabolic control, and metabolic disorders can accelerate periodontal destruction [4,5,6]. People with poor periodontal health are more likely to develop components of MetS and to have adverse cardiometabolic outcomes [4,5]. The association of periodontal diseases with MetS and obesity has been reviewed in detail elsewhere [7].

Digital technologies are increasingly used to integrate complex clinical information and to improve patient care across medical specialties.

Over the past decade, smart technologies have been adopted for the diagnosis, prevention and management of both MetS and oral diseases. In this review, artificial intelligence (AI) refers broadly to computational systems that perform tasks normally requiring human judgement; machine learning (ML) is the subset of AI in which algorithms learn patterns from data; and deep learning (DL) is a subset of ML that uses multilayer neural networks. The Internet of Things (IoT) denotes networks of connected devices that collect and exchange data, and the Internet of Medical Things (IoMT) its application in healthcare. “Smart technologies” is used here as an umbrella term for these approaches, together with wearable devices, telemedicine, mobile health (mHealth) applications and intraoral sensors.

Reviews of artificial intelligence in dentistry have not addressed metabolic disease [8,9,10], and machine learning models for MetS prediction have been developed largely without oral health data [11,12,13]. We did not identify a review examining how smart technologies could connect the assessment and management of these two conditions. This gap is clinically relevant: periodontitis and MetS share risk factors and inflammatory pathways, yet they are assessed by different professionals, in different settings, and recorded in separate information systems, so opportunities for earlier detection of either condition may be missed. Unlike previous reviews, the present work examines both fields with the same critical lens, classifies each application by its current level of evidence and separates what has been demonstrated for combined oral–metabolic approaches from what remains conceptual. This narrative review therefore addresses three questions: (1) Which smart technologies are currently used to predict, monitor and manage MetS? (2) Which are used in oral diagnosis and care? (3) What evidence supports combining oral and metabolic data, and what is still missing?

## 2. Materials and Methods

This narrative review was conducted in accordance with the Scale for the Assessment of Narrative Review Articles (SANRA) [14]. A narrative format was chosen because of the broad scope of the topic and the heterogeneity of study designs in the available literature. SANRA was used as a quality framework rather than as a reporting protocol: it guided the justification of the article’s importance, the statement of specific aims, the description of the literature search, appropriate referencing, scientific reasoning and the presentation of relevant outcome data. This review is not a systematic review. The protocol was not registered, the search was not designed to be exhaustive, no formal risk-of-bias tool was applied, and no quantitative synthesis was attempted. The structured search and the documented selection process described below were used to make the selection of the literature more transparent, not to claim completeness.

A literature search was conducted in PubMed, Scopus and Web of Science for articles published between January 2021 and May 2026. The search combined two concept blocks: condition terms (metabolic syndrome or oral and periodontal health), searched in titles, abstracts and subject headings, and technology terms (artificial intelligence, machine learning, deep learning, wearable devices, Internet of Things, telemedicine and telehealth, mobile health and mobile applications, tele-dentistry, biosensors, intraoral sensors and remote monitoring), searched in titles only, so that the technology was the main subject of the retrieved records. Medical Subject Headings (MeSH) were used in PubMed and equivalent free-text terms in Scopus and Web of Science, linked with Boolean operators (OR within blocks, AND between blocks). Filters were applied for publication date (1 January 2021 to 31 May 2026), English language and document type (articles and reviews; in PubMed, editorials, letters and comments were excluded). Because Scopus can only be limited by publication year, Scopus records published after 31 May 2026 were removed during screening. The complete search strings are reported in Appendix A (Table A1). All three databases were searched on 26 September 2026. Seminal methodological or landmark publications outside this window were cited where needed for context.

The inclusion criteria were (1) original studies (cross-sectional, case-control or cohort) and systematic reviews or meta-analyses; (2) studies on the application of smart technologies in MetS or oral health; and (3) full text available in English.

Exclusion criteria included (1) duplicate publications; (2) animal or in vitro studies, except those directly informative about mechanistic pathways; and (3) conference abstracts without accessible full text.

The searches identified 6922 records (PubMed 2482; Scopus 2490; Web of Science 1950). Duplicates were identified automatically by matching the digital object identifier, the PubMed identifier or the normalized title together with the publication year; 3402 duplicates were removed, leaving 3520 unique records. Of these, 1668 were excluded using predefined keyword rules applied to titles and abstracts by an AI tool (Claude Opus 5.5, Anthropic PBC, San Francisco, CA, USA), for example, records on forensic odontology, dental education, dental materials, in vitro studies or non-systematic reviews. The authors screened the remaining 1852 records by title and abstract and, consistent with the narrative design, selected studies for full-text reading and discussion according to their relevance to the three review questions, giving priority to systematic reviews, large cohort studies and studies reporting validated models or clinical outcomes; records were not screened exhaustively against a formal full-text protocol. Of the 97 publications cited in this review, 31 were retrieved by the database search, and 66 were identified through reference lists of included articles and targeted searches for methodological, regulatory and landmark sources. Of the 31, 24 came from the screened records and 7 from records that had been excluded by the keyword rules, including 1 eligible systematic review, which shows that the automatic exclusion was not free of error. The selection process is summarized in Figure A1 (Appendix A). Because studies were selected by relevance rather than through independent duplicate screening, inter-rater agreement was not calculated. The characteristics of the key studies are summarized in the tables in Section 3.1 and Section 5.4. Relevant data were extracted and synthesized narratively, organized by theme (smart technologies in metabolic syndrome, in oral health and in both). Reference lists of included articles were also screened to identify relevant studies not captured by the database search.

A formal risk-of-bias assessment was considered beyond the scope of this narrative review because the included evidence spans prediction-model, interventional, diagnostic-accuracy and engineering studies, each of which requires a different appraisal tool. Instead, each key study was appraised qualitatively against the main domains of design-appropriate tools: for prediction models, the participants, predictors, outcome and analysis domains of PROBAST, including overfitting, handling of predictors and type of validation, as well as the corresponding TRIPOD+AI reporting items (Section 7); for intervention studies, randomization, the comparator, follow-up and whether the effect of the technology could be separated from that of the accompanying lifestyle program; and for diagnostic studies, the reference standard, the population spectrum and the reporting of sensitivity, specificity and agreement. These appraisals informed the classification of applications by level of evidence (Section 7), and the main risk-of-bias signals for the prediction-model studies are summarized in Appendix A (Table A2); the conclusions were formulated cautiously in light of this limitation.

Systematic reviews were used to summarize the overall direction of the evidence and primary studies to illustrate specific designs, populations or results. Because no quantitative synthesis was performed, overlap between reviews and the primary studies they include could not inflate pooled estimates; however, it could give an impression of more consistent evidence than exists. Where a cited primary study was likely to be included in a cited review, it was therefore not counted as independent confirmation of that review (for example, in Section 5.3), and performance figures were taken from the primary studies rather than from secondary summaries.

## 3. Smart Technologies for the Prediction, Monitoring and Management of Metabolic Syndrome

### 3.1. Machine Learning for the Prediction of MetS

ML has become an important tool in predictive medicine. It can analyze large and complex clinical, genetic and behavioral datasets, identify underlying patterns and estimate an individual’s risk of MetS before clinical onset [15]. ML-based prediction models could therefore support population screening, prevention and personalized medicine by identifying high-risk individuals early.

Several ML algorithms have been used to predict MetS. Widely used classification algorithms include logistic regression (LR), decision trees (DTs), random forest (RF), support vector machines (SVMs) and k-nearest neighbors (kNN) [16]; these methods classify individuals into low-risk and high-risk groups for MetS. In a study of 4756 participants, random forest showed the best overall performance (area under the receiver operating characteristic curve [AUC] 0.804), identifying body mass index (BMI), physical activity and age as the most influential predictors [11].

Ensemble methods combine several models to improve predictive performance; widely used examples include XGBoost, gradient boosting, AdaBoost and LightGBM [17]. In a study enrolling 2400 patients, XGBoost performed best, with an accuracy of 88.97% and an F1 score of 0.913, with waist circumference being identified as the most important predictor [15].

Models based on artificial neural networks (ANNs) and deep neural networks (DNNs) are used to capture complex associations between variables. A multinational study developed prediction models in the Chinese CHARLS cohort and validated them in three external cohorts (KNHANES, UK Biobank and NHANES); the best-performing algorithm differed between cohorts (multilayer perceptron, logistic regression or XGBoost), with AUC values of 0.856 to 0.910 [12].

ML models for MetS use several types of variables.

The core predictors are BMI, waist circumference, blood pressure, triglycerides, HDL cholesterol and blood glucose. Because these variables also define MetS, it is not surprising that they show the strongest predictive value [18].

Frequently used behavioral predictors include physical activity, alcohol consumption, smoking and dietary energy intake.

ML models have been used both to predict MetS and to estimate how lifestyle changes affect its development. Algorithms such as random forest and SVMs have been applied to sociodemographic, clinical and laboratory data to predict the onset of MetS. Models trained on health check-up data have performed well and have also been used to simulate how lifestyle changes would alter individual predictions; the authors recommended external validation before such models are used to identify at-risk individuals and support prevention [19].

For example, a 2% reduction in BMI combined with increased physical activity was estimated to lower the probability of MetS by about 3.8% [19].

Recent models also include single nucleotide polymorphisms (SNPs), polygenic risk scores (PRSs) and metabolic biomarkers [20].

In a 14-year prospective cohort, combining genetic and clinical data in random forest and AdaBoost models yielded an AUC of 0.994 for incident MetS [13]. This near-perfect value was obtained with internal validation only and should be regarded as an optimistic estimate until it is reproduced in other populations.

The main advantages of ML for predicting MetS are its ability to analyze large and heterogeneous datasets (clinical, genetic and lifestyle data), to capture non-linear relationships that conventional statistical methods may miss, to generate individualized predictions that support personalized care and to identify high-risk individuals before clinical symptoms manifest [15].

Several limitations remain: a lack of standardized datasets, risk of bias and limited generalizability, the low interpretability of complex models such as deep neural networks and the need for rigorous clinical validation before widespread use.

Moreover, many studies rely on local databases, which limits the external validity and transferability of the resulting models [15].

High discrimination does not by itself indicate clinical usefulness. First, apparent performance can be inflated by overfitting, particularly when many candidate predictors and hyperparameters are tuned in modest samples, and by data leakage, for example, when imputation, feature selection or oversampling is performed before the data are split or when repeated visits from the same individual appear in both training and test sets. Second, internal validation (cross-validation, bootstrapping or a random hold-out set) estimates performance in data drawn from the same source, whereas external validation in an independent population, setting or time period is required to assess transportability. Third, discrimination, as measured by the AUC, shows only whether a model ranks individuals correctly; calibration, the agreement between predicted and observed risk, and clinical utility, for example, net benefit across decision thresholds, determine whether predictions can guide clinical decisions (Section 7). Fourth, the studies differed in population, MetS definition (NCEP ATP III, IDF or harmonized criteria), predictor sets and validation procedures, so their AUC values are not directly comparable. Finally, models that include the defining components of MetS (waist circumference, triglycerides, HDL cholesterol, blood pressure and fasting glucose) risk circular prediction: when the outcome is prevalent MetS assessed at the same visit, such models largely reproduce the diagnostic rule. Models that predict incident MetS in individuals free of the syndrome at baseline are more informative for prevention [13,19].

Several studies in Table 1 show concrete signals of optimistic performance. In the study by Pawade et al., accuracy fell from 95% in the training data to 88.97% in the test set, a gap typical of overfitting [15]; the 100% test accuracy reported by Boitor et al. (Table 1) was obtained in a test set of about 89 patients from a 70/30 split of 296; and the AUC of 0.994 reported by Shin [13] came from models that included a genome-wide polygenic score and were validated internally only. Further sources of inflation include selecting the best of many algorithms or automated machine learning pipelines on the same test data, which rewards chance variation; reporting accuracy, which depends heavily on the prevalence of MetS in the sample; oversampling or other resampling performed before the data are split; and, in longitudinal data such as the 37,999 visit pairs from 17,182 individuals analyzed by Tavares et al. [19], leakage between training and test sets unless the split is made at the level of the individual rather than the visit.

Table 1 summarizes the design, validation and reported performance of the aforementioned MetS prediction studies.

### 3.2. Wearable Devices and the Internet of Things

Over the past decade, wearable sensors and IoT platforms have become important tools for the continuous assessment of physiological parameters and the prevention of metabolic disorders.

Wearable devices record physiological data in real time, including heart rhythm, physical activity, sleep and glucose levels. When integrated with IoT platforms, these data can be processed and sent to clinicians or to AI algorithms for assessment of metabolic risk [23].

By collecting physiological data continuously and linking them to analytical platforms, these technologies can help to identify metabolic risk early and to tailor interventions. Their role in preventive care and chronic disease management is likely to grow as sensors become more accurate and algorithms more sophisticated.

Wearable devices used to monitor metabolic health include smartwatches, activity trackers, biometric patches and dedicated sensors for electrocardiography (ECG) or continuous glucose monitoring (CGM), as well as smart rings and textile-embedded sensors.

A systematic review of 53 studies found that smartwatches were the most frequently used devices (45.3%), followed by biometric patches (34%), chest belts (22.6%) and fitness bracelets (13.2%) [23].

These devices track physiological parameters relevant to the assessment and management of metabolic health.

Wearable devices can continuously record cardiovascular parameters, such as heart rate (HR), heart rate variability (HRV) and electrocardiogram (ECG) data, and metabolic indicators, such as glucose, lactate, sweat biomarkers and body temperature; behavioral parameters can also be recorded, such as physical activity, sleep duration and quality, energy expenditure and stress. Elevated heart rate measured by wearable devices has been associated with a higher risk of MetS, suggesting a role in early risk identification [24]. Wearable biochemical sensors can also measure sweat biomarkers, offering a non-invasive way to track metabolic indicators [25].

Continuous monitoring of behavior is important because lifestyle strongly influences the development of MetS.

The Internet of Medical Things (IoMT) is a network of connected devices that collect health data and transmit them to digital platforms or health information systems.

A typical system includes wearable sensors that collect physiological data, a gateway (such as a smartphone or hub) that relays them and a cloud infrastructure for storage and analysis [26].

Such systems allow for continuous remote monitoring of patients and reduce the need for frequent clinic visits.

Combining wearable devices with IoT platforms enables continuous monitoring of people at risk of metabolic disorders, automatic transmission of data to healthcare professionals, early detection of abnormalities and timely lifestyle interventions [27].

In a small prospective pilot study of patients with type 2 diabetes, use of a wearable device connected to an internet management platform was associated with improved glycemic control and treatment adherence [28]; because the population did not have MetS specifically and the device was used within a broader management program, the effect cannot be attributed to the device alone.

Clinical applications of wearables and IoT in MetS are summarized in Figure 1.

The main advantages of wearable and IoT technologies are continuous monitoring, automatic data collection, early identification of metabolic risk, individualized evidence-based interventions and potentially lower healthcare costs [32].

These platforms can also be linked to ML algorithms to predict the progression of metabolic disease.

Limitations include variable sensor accuracy, data privacy and security concerns, a lack of device standardization and the need for further clinical validation.

The effect of wearable devices on cardiometabolic markers varies between studies, and further research is needed to establish their clinical value [23].

### 3.3. Telemedicine and Mobile Health

Telemedicine and mHealth applications support the monitoring, prevention and treatment of chronic diseases, including MetS. They enable continuous monitoring of physiological indicators, virtual contact between patients and clinicians and the behavioral changes needed to reduce cardiometabolic risk [33].

Telemedicine enables remote patient monitoring through digital platforms, connected devices and virtual consultations, which is particularly useful for chronic conditions that require long-term follow-up. Its potential benefits include regular monitoring of blood pressure, glucose, lipids and anthropometric measures; fewer in-person visits; better adherence to treatment and lifestyle advice; continuous patient education; and lower costs for health systems and patients [34].

In some telemedicine programs for MetS, patients monitored remotely achieved better control of blood pressure and cholesterol than those receiving conventional care and reported high satisfaction with the digital service [35].

Remote rehabilitation and digital monitoring can also support changes in diet and physical activity, which are central to the treatment of MetS.

Mobile applications are widely used to manage the risk factors of MetS, including obesity, hypertension, dyslipidemia and hyperglycemia. Their core functions include tracking body weight and BMI, recording dietary intake, monitoring physical activity, sending medication reminders and providing personalized feedback and virtual coaching [36].

A 12-week home-based, telemonitored training program in patients with MetS was followed by significant reductions in body weight, waist circumference, fasting plasma glucose and glycated hemoglobin (HbA1c), together with increases in HDL cholesterol and exercise capacity [37]; this was a single-group study, so the changes cannot be attributed to telemonitoring itself.

Home monitoring of blood glucose, blood pressure and physical activity through mobile applications linked to connected sensors may allow for more individualized interventions. For instance, in a three-arm randomized controlled trial of adults with MetS, a lifestyle program supported by a mobile application produced greater reductions in body weight, waist circumference, body mass index and systolic blood pressure than the same program supported by a booklet [38].

Systematic reviews and meta-analyses have also associated app-based digital interventions with improvements in BMI, waist circumference, blood pressure, glycemia and HDL cholesterol [35].

Taken together, the evidence for wearables, telemedicine and mHealth in MetS should be interpreted with caution. Most interventions combined a device or application with structured lifestyle counselling, exercise training or clinician feedback, so the contribution of the technology itself is difficult to isolate; designs that deliver the same program with and without the digital component, such as the three-arm trial comparing a mobile application with a booklet [38], are needed for this purpose. The populations studied ranged from employees with MetS [31] and patients with type 2 diabetes [28] to adults with established MetS [37,38], so findings from one group cannot be generalized to all individuals with MetS. Follow-up was often 12 weeks or shorter, and several changes, although statistically significant, were small in absolute terms. Whether such changes are clinically meaningful, for example, against commonly used thresholds such as a weight loss of at least 5% or blood pressure reductions large enough to lower cardiovascular risk, and whether they persist after the intervention ends remains uncertain [23,35].

## 4. Smart Technologies in Oral Health

### 4.1. Artificial Intelligence in Dental Diagnosis

In dentistry, AI is used to interpret radiographs, detect oral diseases early and support diagnosis and treatment planning. Machine learning and deep learning algorithms, especially convolutional neural networks (CNNs), allow for rapid analysis of dental images and accurate identification of abnormalities [8].

CNNs can automatically detect dental caries, periodontal disease and other radiographic abnormalities.

For example, Kim et al. (2025) developed a CNN-based model for remote caries detection that uses Grad-CAM heat maps to show which image regions drive each prediction [39].

AI therefore has considerable potential in dental diagnosis, treatment planning and prevention and can also support periodontal treatment planning [40].

AI can analyze large clinical and imaging datasets, which may enable faster and more accurate diagnosis. AI systems can identify subtle patterns in radiographs or intraoral photographs that may be missed by the human eye [9].

The main underlying technologies are ML, in which algorithms improve their performance by learning from data; DL, which uses multilayer neural networks suited to image interpretation; and computer vision, which enables automated analysis of radiological images [9].

Using these methods, AI systems can detect early signs of dental caries, periapical lesions, periodontal bone loss and oral tumors [41]. Key applications are summarized in Figure 2.

The reported advantages of AI in dental diagnosis include high diagnostic accuracy, earlier detection of oral disease, fewer human errors, faster processing of radiographs and better support for clinical decisions.

AI may also improve workflow efficiency in dental practice and support a more predictive and personalized approach to care [46].

AI also has limitations, including variable quality of training data, the risk of errors when datasets are incomplete, a lack of standardized models and ethical and legal issues related to accountability and data protection.

AI should therefore be seen as a tool that supports the clinician, not as a substitute for clinical judgement [10]. Most of the evidence comes from retrospective diagnostic-accuracy studies on curated image sets; prospective evaluations in routine practice and studies showing improvements in patient outcomes remain scarce [46]. Performance figures from such studies are also vulnerable to domain shift: models trained on images from one center, device or population often perform worse elsewhere, and screening in community settings, with smartphone photographs taken by non-specialists, poses different problems from the analysis of curated radiographs. External validation and community deployment are discussed in Section 7.

### 4.2. Tele-Dentistry

Tele-dentistry is the use of telecommunication and digital technologies to deliver dental care remotely. It includes virtual consultations, diagnosis, treatment monitoring and patient education, delivered through web platforms, mobile applications, digital imaging and video calls. It is a branch of telemedicine and has become important for improving access to oral healthcare, especially for people in remote or underserved areas [47].

Tele-dentistry dates back to the 1990s, when it was first used to facilitate communication between general dentists and specialists in different locations. Using digital tools, clinicians can assess radiographs and intraoral photographs and give expert advice without the patient being present [48].

Tele-dentistry relies on high-speed internet connections, videoconferencing systems, protocols for transmitting digital dental images and patient monitoring platforms.

These technologies allow dental care to be delivered synchronously (in real time) or asynchronously (through review of stored patient data). The main methods and applications are summarized in Table 2.

Tele-dentistry offers several advantages, including better access to oral healthcare, lower travel costs and time, early detection of oral disease, continuous patient monitoring and easier collaboration between specialists [53].

During the COVID-19 pandemic, tele-dentistry helped to maintain oral healthcare while limiting the spread of infection [54]. Remote education, photographic assessment and preliminary screening tools have reduced unnecessary in-person visits and can improve adherence to oral hygiene, particularly in patients with other medical conditions [55].

Tele-dentistry also has limitations: treatment cannot be delivered remotely, diagnosis depends on the quality of transmitted images, patient data must be protected, a reliable technical infrastructure is required, and patients vary in digital literacy [56].

Tele-dentistry is therefore best regarded as a complement to conventional dental care.

### 4.3. Intraoral Sensors and Biomarkers

Intraoral sensors and biomarkers are modern tools for assessing oral health and for the early detection of oral and systemic diseases. They allow for rapid diagnosis, continuous monitoring of the oral environment and individualized treatment. Recent advances in nanotechnology, biosensors and molecular analysis have accelerated their development.

Intraoral sensors are miniaturized devices placed in the mouth to measure physiological, chemical or physical parameters. They can be integrated into orthodontic appliances, oral splints, removable prostheses or directly onto tooth surfaces [57].

Biomarkers are measurable biological indicators that reflect normal physiological states, disease processes or responses to treatment.

In dentistry, biomarkers are measured mainly in saliva, gingival crevicular fluid, blood and oral tissue samples. The main types of intraoral sensors and biomarkers are summarized in Table 3.

These methods could offer early detection of oral disease, continuous monitoring of oral health, personalized treatment, minimally invasive assessment and, through saliva analysis, the possibility of detecting systemic disease.

They also support personalized dental care, in which treatment is adapted to the patient’s individual biological profile.

Limitations include the high cost of advanced technologies, the need for large clinical trials, concerns about material biocompatibility and durability and difficulties in integrating these tools into routine dental practice. Apart from established laboratory assays, most of the sensors and biomarker panels in Table 3 remain at the stage of laboratory or pilot validation (Section 7) [57,71].

Advances in nanotechnology and AI are expected to further improve intraoral sensors and biomarker detection systems.

Sensors that track salivary pH and inflammatory markers could allow for oral health data to be incorporated into broader systemic prediction models. Experimental intraoral sensors that measure pH, the oral microbiome or gingival inflammation are under development. Linked to smartphones, they could provide data for models that predict periodontal disease and its relationship with systemic inflammation [71].

## 5. Evidence for Integrating Oral and Metabolic Data

### 5.1. Associations Between Periodontal Indicators and MetS

Chronic low-grade inflammation is the main biological link between periodontitis and MetS. Inflamed periodontal tissues are a persistent source of inflammatory mediators and bacterial products, while visceral adiposity and insulin resistance in MetS sustain a pro-inflammatory state that can accelerate periodontal destruction [4,5,6,7]. Population data are consistent with this link. In 3996 adults aged 35–70 years from the Kurdish Dehgolan cohort, MetS (NCEP ATP III) was more common among participants who did not brush or floss daily and among those with missing teeth [72]. For data-driven models, this means that routinely recorded dental variables, such as periodontal indices, tooth loss and oral hygiene behavior, are candidate predictors of metabolic risk and that metabolic variables are candidate predictors of periodontal risk. These observations, however, are largely cross-sectional: they establish association, not causality, and do not by themselves show that oral variables add predictive value beyond established metabolic risk factors.

### 5.2. Prediction Models Using Combined Oral and Medical Data

A small number of studies have applied machine learning to combined dental and medical data. Boitor et al. studied 296 hospitalized patients aged 45–79 years in Sibiu, Romania, 57% of whom had MetS, and combined periodontal and dental indices (decayed, missing and filled teeth [DMFT], Community Periodontal Index [CPI], pocket depth, attachment loss and bleeding on probing), oral hygiene habits, cardiovascular risk, carotid atherosclerosis and quality of life (EQ-5D-5L) [22]. Using automated machine learning with SHapley Additive exPlanations (SHAP), they found that the severity of periodontal disease, high cardiovascular risk and a low EQ-5D-5L score had the greatest influence on the presence of MetS. Because the study was cross-sectional, hospital-based and evaluated with a single train–test split in fewer than 300 patients, the reported 100% test accuracy (Table 1) most likely reflects overfitting and should not be interpreted as diagnostic performance.

Other studies have modeled the relationship in the opposite direction. Wilensky et al. analyzed 57,496 linked dental and medical records of Israeli military personnel from the DOME study and used XGBoost to rank the systemic conditions associated with periodontitis; obesity, obstructive sleep apnea and non-alcoholic fatty liver disease were the most important features [73]. In all of these studies, oral and metabolic data were collected at the same time point, and the incremental value of oral variables over established metabolic predictors remains to be quantified in independent data. Vu et al. applied several machine learning classifiers to NHANES 2009–2014 data to predict periodontal disease in US adults [74], and artificial intelligence is increasingly used to detect and stage periodontal disease from clinical and radiographic data [75].

These studies also show the limits of the approach. Soysal and Öner compared classical regression with random forest and deep learning models for the association of HbA1c and BMI with periodontal outcomes in 1852 NHANES participants [76]. The random forest models produced unstable and clinically implausible risk patterns, whereas probability-based models showed a consistent rise in periodontal risk with increasing HbA1c; the authors concluded that, for modest and mainly linear metabolic–periodontal relationships, traditional statistical models remain more robust and easier to interpret. Machine learning is therefore most useful when many heterogeneous predictors interact, and it should be compared against a well-specified regression model rather than assumed to be superior.

Read together, the combined studies point in a consistent direction, although the evidence is thin. In the studies that examined oral variables as correlates of MetS, worse periodontal status (greater pocket depth, attachment loss or tooth loss) and poorer oral hygiene were associated with a higher likelihood of MetS [22,72]; conversely, metabolic conditions were associated with a higher likelihood or greater severity of periodontitis [73,76]. Among the MetS components, adiposity (obesity, BMI) and glycemia (HbA1c) were the factors most consistently linked with periodontal outcomes [73,76], whereas the separate contributions of dyslipidemia and blood pressure were rarely reported. The size of these associations cannot be compared across studies because the populations (hospitalized patients, military personnel and population surveys), the definitions of MetS and periodontitis and the analytic methods differed, and all of the studies were cross-sectional. The consistency of direction therefore supports biological plausibility, but not causality or added predictive value.

### 5.3. Diagnostic Value of Salivary Biomarkers

Saliva can be collected non-invasively during dental visits and contains markers relevant to both oral and metabolic health (Section 4.3). Salivary glucose is the most studied example. A scoping review of 57 studies in type 1, type 2 and gestational diabetes found consistent positive correlations between salivary and blood glucose but wide variation in sensitivity, specificity and analytical methods [77]. In a cross-sectional study of 166 people with and without diabetes, the correlation was weak in healthy individuals, and the authors concluded that saliva is better suited to monitoring than to diagnosis, particularly in poorly controlled diabetes [78]. Because this study may be among those included in the scoping review [77], it is not counted here as independent confirmation of the review. Sensor development is progressing: an optical salivary glucose biosensor showed correlation coefficients with a commercial glucometer of 0.97 (fasting) and 0.90 (postprandial) in patients with diabetes but only 0.58 and 0.87 in participants without diabetes [79]. Correlation, however, is not agreement: a high correlation coefficient can coexist with large systematic or proportional differences between salivary and blood values, and it does not establish diagnostic equivalence or clinical interchangeability. Moreover, the reference in this study was a commercial glucometer rather than a laboratory method. To support screening, salivary tests would need to report sensitivity, specificity and predictive values against an accepted reference standard (fasting plasma glucose, HbA1c or an oral glucose tolerance test), agreement analyses such as Bland–Altman limits and test–retest reproducibility under standardized collection conditions in the population in which they are intended to be used [77].

Taken together, these data suggest that salivary glucose could support longitudinal monitoring in people with known dysglycemia but not screening in the general population. Combining several salivary markers, for example, glucose with inflammatory cytokines (Table 3), and interpreting them with machine learning is a plausible next step, but this approach has not yet been validated in people with MetS.

### 5.4. Integrated Dental–Medical Pathways: A Conceptual Framework

Table 4 summarizes the studies that inform the integration of oral and metabolic data, grouped by the type of evidence they provide. These categories represent different levels of evidence and are not interchangeable: an association does not demonstrate predictive value, a correlation does not demonstrate diagnostic accuracy, and neither demonstrates that an integrated pathway improves detection, management or patient outcomes. No study in the last category was identified.

In a hypothetical integrated pathway, which has not yet been evaluated, dental visits could serve as an additional point of contact for metabolic screening, and medical visits as a point of referral for periodontal assessment. A patient with severe periodontitis and no known metabolic diagnosis could be flagged for measurement of waist circumference, blood pressure and glucose, while a patient with MetS could be referred for a periodontal examination. Wearable and remote monitoring data (Section 3.2) and salivary sensors could then support follow-up, and machine learning models could combine these inputs into personalized recommendations on diet, physical activity and oral hygiene [80].

The main obstacle is structural rather than technical. Dental and medical records are usually held in separate systems, and few datasets contain high-quality periodontal and metabolic measurements for the same individuals. We did not identify any prospective study that has tested an integrated dental–medical screening pathway supported by smart technologies; this is the main gap identified by this review. Figure 3 presents this as a conceptual framework that requires prospective validation, indicating for each component whether it is supported by published studies, by preliminary or indirect evidence only or not yet evaluated. Section 7 examines the framework against the barriers documented in earlier attempts to integrate oral health into general healthcare.

## 6. Ethical, Practical and Equity Considerations

Using smart technologies in clinical practice raises challenges related to data privacy, the consistency of algorithms, validation across clinical centers and cost.

ML requires large datasets and patient populations to produce reliable results, and AI technologies need to be standardized. International guidelines for medical AI will be needed as the field develops.

Data confidentiality is a further ethical concern. An analysis of top-rated medicine-related apps in the UK, US, Canada and Australia found that sharing of user data was routine and far from transparent [81].

The adoption of AI technologies in clinical practice has been slow, and their costs are substantial [82].

In primary care, a major limitation of ML algorithms is the lack of clear explanations for their diagnostic and therapeutic recommendations [83].

Explanations of algorithm outputs that are understandable to both clinicians and patients would be an important step towards safe clinical use, particularly given concerns about safety, regulatory compliance and equitable access to care [84].

Integrating dental and medical data raises additional specific issues. First, we consider interoperability: dental records are often kept in standalone practice management software that uses coding systems different from those of medical electronic health records, so combined models require common data standards, such as standardized periodontal diagnostic codes and interoperable exchange formats (for example, HL7 FHIR), as well as data-sharing agreements between dental and medical providers. Second is data governance and consent: linking oral and metabolic data creates a more sensitive health profile, so patients should be told which data are shared, with whom and for what purpose, and sharing should rely on secure, privacy-preserving infrastructure that complies with applicable law, such as the General Data Protection Regulation in the European Union.

Third, algorithmic bias must be explored: models developed in single, often hospital-based or regionally restricted, cohorts may perform differently in other ethnic, age and socioeconomic groups, and the MetS definition itself relies on population-specific waist circumference thresholds [3]; performance should therefore be reported, and externally validated, across demographic and clinical subgroups. Fourth, digital literacy and access are important: older adults, people with low income and residents of rural areas, groups in which both MetS and periodontitis are common, are often less able to use smartphones, wearables and online services, so digital pathways may widen inequalities unless non-digital alternatives are maintained [56]. Finally, costs, workflow and professional responsibility must be considered: before an integrated pathway is implemented, it must be clear who acts on an algorithm-generated alert of metabolic risk in a dental setting, how the patient is referred and followed up, how dental professionals are trained and reimbursed for this role and how responsibility is shared between dentists, physicians and software providers [82,83].

## 7. Discussion

The present review examined smart technologies in three areas: the prediction, monitoring and management of MetS, oral diagnosis and care and the combination of the two. Machine learning models for MetS prediction are numerous and often report high discrimination; wearable, telemedicine and mobile interventions have shown benefits for body weight, blood pressure and glycemic markers, while AI tools for dental imaging have shown diagnostic accuracy comparable to that of clinicians, mostly in retrospective studies, with limited evidence of improved patient outcomes. In contrast, work that combines oral and metabolic data is scarce. We identified a few cross-sectional modeling studies and early-stage salivary sensors but no prospective evaluation of an integrated pathway.

Table 5 classifies the applications discussed in this review by their current level of evidence to distinguish technical feasibility from demonstrated clinical effectiveness.

Earlier reviews have addressed these fields separately. Reviews of AI in dentistry have focused on imaging and oral diagnosis [8,9,10], reviews of the periodontitis–MetS association have focused on epidemiology and mechanisms rather than technology [7], and studies of MetS prediction have rarely included oral variables [11,12,13]. The contribution of the present review is to bring these strands together and to identify the specific evidence gaps between them.

Several features of the evidence limit the strength of these conclusions. Most studies were cross-sectional or retrospective, and most prediction models were evaluated only with cross-validation or internal data splits. Of the studies included in Table 1, only Li et al. clearly reported validation in independent external cohorts [12]. Performance close to perfect, such as the AUC of 0.994 reported for a model combining a genome-wide polygenic score with demographic and dietary data [13], should be interpreted with caution until it is reproduced in other populations; with a large number of genetic predictors and internal validation only, overfitting is a plausible explanation. MetS definitions also differed between studies, which affects both prevalence and model performance. Finally, several models used the diagnostic components of MetS, such as waist circumference, triglycerides, HDL cholesterol, blood pressure and glucose, as predictors [15,18]. Such models partly reproduce the diagnostic definition rather than predict disease. Models that predict incident MetS in people free of it at baseline [13,19] are more informative for prevention, whereas models that rely only on simple, non-laboratory measures [21] may be more practical for screening, although they still include anthropometric and blood pressure components of the definition. Prediction of prevalent and of incident MetS answers different clinical questions, and the two should not be compared directly. Future prediction studies should follow the TRIPOD+AI reporting guideline [85] and can be appraised with PROBAST [86]. Judged informally against the PROBAST domains, most models in Table 1 would probably be rated at high or unclear risk of bias in the analysis domain, mainly because of internal-only validation, limited reporting of calibration and incompletely described handling of predictors and missing data. Calibration should be reported alongside discrimination [87], and clinical utility can be assessed with decision-curve analysis [88].

Implementation raises further issues. In addition to the issues discussed in Section 6, regulatory requirements apply: in the European Union, AI software intended for diagnosis or risk prediction generally falls under the Medical Device Regulation [89] and, as a high-risk AI system, under the Artificial Intelligence Act [90], which add requirements for data quality, transparency and human oversight. These frameworks have direct consequences for clinical implementation. Under classification Rule 11 of the MDR, most software that provides information used for diagnostic or therapeutic decisions is in class IIa or higher, so it requires conformity assessment by a notified body, a certified quality management system, clinical evaluation and post-market surveillance [89]. Limited notified-body capacity led to the extension of the MDR transition period for devices certified under the former directives to 2027–2028 [91], and certification of new software can take considerable time. For AI-based medical devices, the high-risk obligations of the AI Act will apply from 2 August 2028, following the amendments introduced by the Digital Omnibus on AI [92]; manufacturers will then have to demonstrate data governance, technical documentation, record-keeping, transparency and human oversight in addition to MDR compliance, and substantial modifications of a model after deployment may require renewed assessment. For research-stage tools such as combined oral–metabolic risk models or salivary sensors, the route to routine use is therefore likely to take several years and sustained investment. Developers should plan regulatory-grade documentation, external validation and post-market performance monitoring from the outset rather than after a successful pilot, and the costs of compliance may favor larger manufacturers over academic groups.

Concrete examples show what external validation adds and why it matters before deployment. For MetS, Li et al. developed models in a Chinese cohort and validated them in Korean, British and US cohorts, and the best-performing algorithm differed between cohorts [12]. In dental imaging, a deep learning model for detecting apical lesions on panoramic radiographs reached an F1-score of 54.1% at the center where it was trained but 32.7% at an external center, and its performance depended on the dental status of the population rather than on image characteristics [93]. Moving from retrospective image sets to community use introduces further shifts in operators, devices, lighting and populations; Chau et al. addressed this by externally validating a smartphone-based mHealth tool for detecting gingival inflammation in community-dwelling older adults [94]. Studies of this kind, rather than internal test-set performance, are the relevant evidence for using AI tools in community or chairside screening.

Experience with chairside diabetes screening in dental settings illustrates both the opportunity and its limits. In a dental clinic in New York, an algorithm based on the number of missing teeth and the percentage of deep periodontal pockets identified patients with unrecognized diabetes or prediabetes, and its performance improved when a point-of-care HbA1c test was added [95]. In a field trial in US dental practices, 416 of 1022 patients aged 45 years or older without known diabetes (40.7%) had an HbA1c of 5.7% or higher and were referred, but only 35.1% of those referred received a diagnosis from a physician within one year, and follow-up was poor among patients of private dental offices [96]. These studies concern dysglycemia rather than MetS and did not use smart technologies, but they show that dental settings can identify metabolic risk and that completion of referral, rather than detection, is often the weakest link.

Tested against these documented barriers, the framework in Figure 3 faces obstacles beyond technology. A scoping review of efforts to integrate oral health into primary care identified low political priority and separate financing of dental and medical care; implementation problems such as cost, workload, staff shortages and missing dental information in medical records; discipline-specific training with limited competencies outside each profession’s own field; weak referral pathways and limited continuity of care; and patients’ own perception of low need [97]. Each maps onto a component of the framework: separate insurance and record systems limit data integration; dentists may lack the training, time and reimbursement to act on metabolic risk; incomplete referral, as in the dental screening trial above [96], would cancel the benefit of detection; and patients may not see a dental visit as the place for metabolic assessment. An implementation study of the framework should therefore measure referral completion, time to diagnosis, provider workload and cost, alongside diagnostic accuracy.

This review is narrative in nature. A formal risk-of-bias assessment was not performed; key appraisal domains were considered qualitatively instead (Section 2), and study selection may still reflect the authors’ judgement. The structured search reduces but does not eliminate selection bias, and the review should not be read as an exhaustive or systematic evidence map. Only English-language publications from January 2021 to May 2026 were searched systematically, and the database searches may not have captured all relevant studies. The included studies differ widely in populations, MetS definitions, technologies and outcomes, which precluded quantitative synthesis, and several of the performance figures cited come from single studies that have not been replicated.

It is important to separate what this review has established from what it proposes. The published evidence shows that periodontal indicators are associated with MetS, that oral variables can be incorporated into prediction models and that salivary glucose tracks blood glucose, mainly in people with diabetes. The framework in Figure 3, including metabolic risk assessment in dental settings and periodontal referral from medical settings, is a proposal for future research: its added diagnostic value, clinical effectiveness, feasibility and cost effectiveness have not yet been established.

Several research priorities follow: First, prospective cohorts should collect standardized periodontal examinations alongside metabolic measurements, so that combined models can be developed for incident rather than prevalent disease. Second, models should be externally validated in independent populations, report calibration as well as discrimination, assess clinical utility and be compared with simple regression models. Third, salivary and intraoral sensors should be validated against laboratory reference methods specifically in people with MetS. Fourth, pragmatic trials should test whether metabolic screening in dental settings and periodontal referral from medical settings improve detection and clinical outcomes. Finally, cost effectiveness and equity of access should be evaluated so that these technologies do not widen existing health inequalities.

## 8. Conclusions

Smart technologies, including ML, wearable and IoT devices, telemedicine and intraoral sensors, offer promising opportunities to support the prediction, monitoring and management of MetS and oral diseases, but the two fields have developed largely in parallel. The evidence supporting the integration of oral and metabolic data remains limited: most available studies are cross-sectional analyses, internally validated prediction models or preliminary sensor studies, and salivary glucose has shown correlation with blood glucose, mainly in people with diabetes, rather than established diagnostic accuracy for screening. Within metabolic risk prediction, models that predict incident MetS in people free of the syndrome at baseline have greater preventive value than models that largely reproduce its diagnostic components because they can identify people at risk, while lifestyle, periodontal and other preventive interventions may still delay or avert its onset; longitudinal cohort studies with standardized oral and metabolic measurements are needed to develop and externally validate such models. The added diagnostic value, clinical effectiveness, feasibility and cost effectiveness of integrated dental–medical pathways have not yet been established, and regulatory approval under the MDR and the AI Act will further shape how quickly validated tools can reach routine care. Future research should prioritize prospective cohorts, independent external validation, standardized outcome definitions and clinical trials evaluating whether such pathways improve diagnostic accuracy, patient outcomes, feasibility and cost effectiveness. Until such evidence is available, these approaches should be considered promising research directions rather than established routine clinical practice.

## Figures and Tables

**Figure 1:**
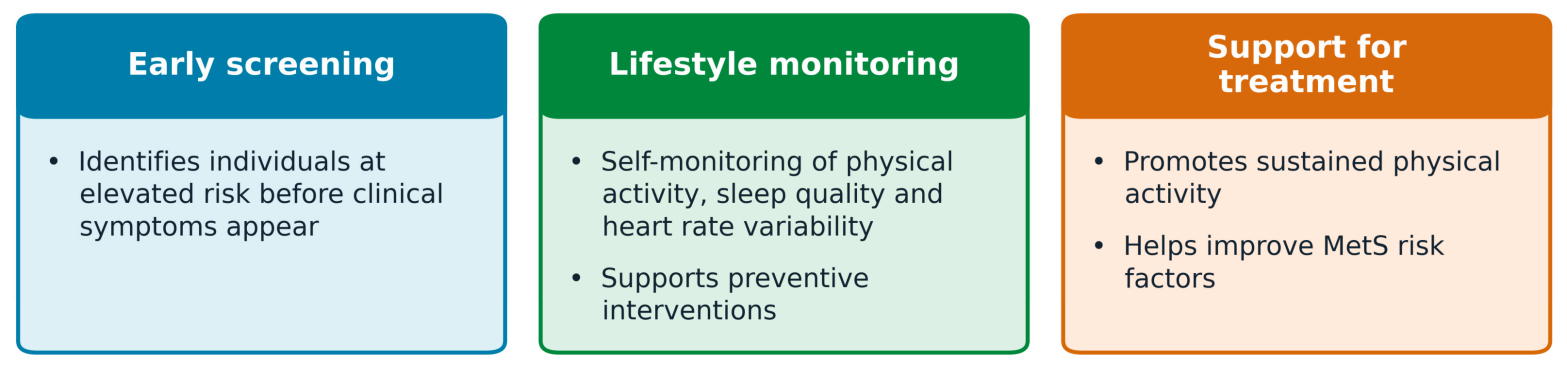
Clinical applications of wearables and IoT in metabolic syndrome. IoT, Internet of Things; MetS, metabolic syndrome [29,30,31].

**Figure 2:**
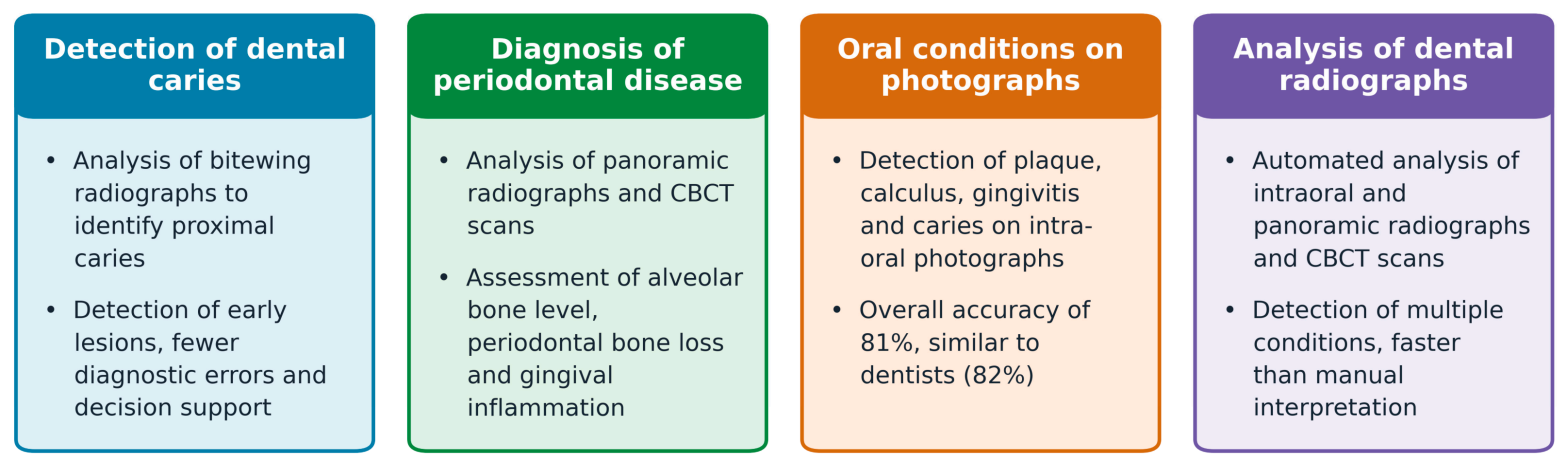
Applications of artificial intelligence in dental diagnosis. CBCT, cone-beam computed tomography [42,43,44,45].

**Figure 3:**
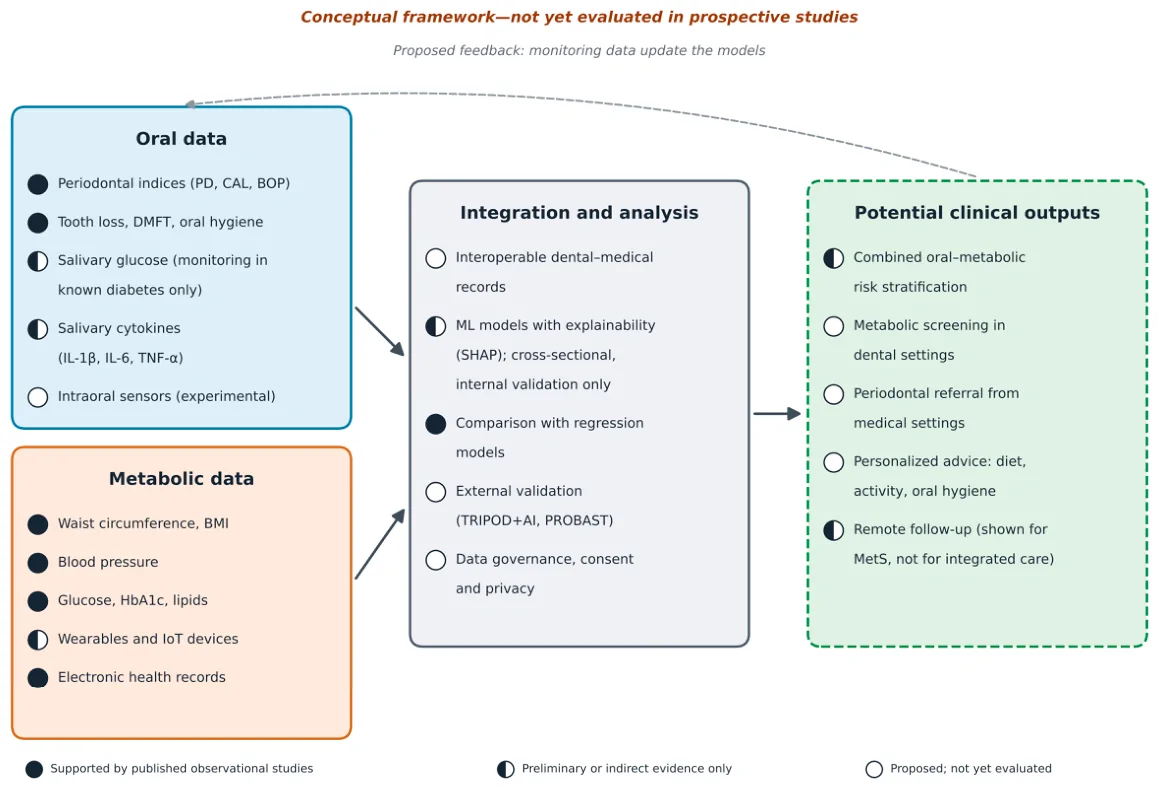
Conceptual framework for integrating oral and metabolic data using smart technologies. The framework is a proposal for future research and has not been evaluated prospectively. Filled circles indicate components supported by published observational studies; half-filled circles, components supported by preliminary or indirect evidence only; open circles, components not yet evaluated. BMI, body mass index; BOP, bleeding on probing; CAL, clinical attachment loss; DMFT, decayed, missing and filled teeth; HbA1c, glycated hemoglobin; IL, interleukin; IoT, Internet of Things; MetS, metabolic syndrome; ML, machine learning; PD, probing depth; PROBAST, Prediction model Risk Of Bias ASsessment Tool; SHAP, SHapley Additive exPlanations; TNF-α, tumor necrosis factor alpha; TRIPOD+AI, Transparent Reporting of a multivariable prediction model for Individual Prognosis Or Diagnosis, updated for artificial intelligence.

**Table 1:** Machine learning studies for metabolic syndrome prediction discussed in this review.

Study	Setting/Data	*n*	Algorithms	Predictors	Validation	Best Reported Performance
Pawade et al., 2024 [15]	NR	2400	Nine classifiers, incl. LR, KNN, SVC, DT, RF, AdaBoost, XGBoost, LightGBM	Incl. waist circumference	Internal (held-out test set)	XGBoost: test accuracy 88.97% (training 95%); F1 0.913
Akbarzadeh et al., 2022 [11]	Iran, Tehran Cardio-metabolic Genetic Study	4756	LR, DT, RF, SVM, discriminant analysis	Demographic, clinical, GCKR variants	Internal (10 × repeated 10-fold CV)	RF: AUC 0.804; accuracy 0.743
Li et al., 2024 [12]	Development: China (CHARLS); validation: KNHANES, UK Biobank, NHANES	6155 (development); 5297/218,781/2549 (validation)	Nine algorithms, incl. MLP, LR, XGBoost	LASSO-selected from 59 candidate variables	External (three cohorts)	AUC 0.856–0.910 (best algorithm varied by cohort)
Mirzaei et al., 2025 [18]	Iran, Ravansar Non-Communicable Disease Cohort (PERSIAN), adults 35–65 years; IDF criteria	9602	Boruta feature selection with ROC analysis	Age, waist and hip circumference, BMI, fasting glucose, SBP–DBP, triglycerides	NR	AUC 0.89 (men); 0.86 (women)
Tavares et al., 2022 [19]	Brazil, check-up program over 17 years	17,182 (37,999 visit pairs)	LR, LDA, KNN, DT, LightGBM, XGBoost	Sociodemographic, clinical, laboratory and lifestyle variables	Internal (authors recommend external validation)	LightGBM: AUC 0.86; sensitivity 87.8%; specificity 70.2%
Shin D, 2024 [13]	Korea, KoGES Ansan–Ansung (incident MetS at 14 years)	5440	Cox, DNN, SVM, SGD, RF, NB, AdaBoost	Demographic, lifestyle, dietary (dried laver), genome-wide PRS	Internal	RF and AdaBoost: AUC 0.994
Shin H et al., 2023 [21]	Korea, KoGES health examinations 2004–2013, adults 40–69 years	70,370	LR, DT, RF, XGBoost, TabNet; DT selected	Waist circumference, blood pressure, sex (no blood tests)	Internal (10% hold-out test set)	DT: AUC 0.889; recall 0.855; specificity 0.773
Boitor et al., 2023 [22]	Romania, hospitalized adults aged 45–79 years	296	AutoML (H2O, Auto-sklearn); best model XGBoost	Periodontal indices, oral hygiene, cardiovascular risk, carotid atherosclerosis, EQ-5D-5L	Internal (70/30 train–test split)	XGBoost: 100% test accuracy; SHAP: periodontal pocket depth, cardiovascular risk and EQ-5D-5L most influential

AUC, area under the receiver operating characteristic curve; BMI, body mass index; CV, cross-validation; DNN, deep neural network; DT, decision tree; KNN, k-nearest neighbors; LDA, linear discriminant analysis; LR, logistic regression; MLP, multilayer perceptron; NB, naïve Bayes; NR, not reported; PRS, polygenic risk score; RF, random forest; SBP–DBP, systolic–diastolic blood pressure; SGD, stochastic gradient descent; SHAP, SHapley Additive exPlanations; SVC/SVM, support vector classifier/machine; AutoML, automated machine learning; CHARLS, China Health and Retirement Longitudinal Study; EQ-5D-5L, EuroQol 5-dimension, 5-level questionnaire; GCKR, glucokinase regulator gene; IDF, International Diabetes Federation; KNHANES, Korea National Health and Nutrition Examination Survey; KoGES, Korean Genome and Epidemiology Study; LASSO, least absolute shrinkage and selection operator; NHANES, US National Health and Nutrition Examination Survey; XGBoost, extreme gradient boosting. Internal validation: performance estimated in data from the same source (cross-validation or random split); external validation: performance estimated in an independent cohort. Shin D [13] and Tavares et al. [19] predicted incident MetS; the other studies classified prevalent MetS. Performance values are those reported by the original authors and are not directly comparable because MetS definitions, predictors and validation strategies differ.

**Table 2:** Methods and applications of tele-dentistry.

**Methods**	
Real-time consultation	- enables real-time, interactive communication between patient and clinician, usually by videoconference - allows the clinician to assess symptoms, give advice or decide whether an in-person examination is needed [49]
Store-and-forward	- asynchronous transfer of clinical data, such as radiographs, intraoral photographs and medical history, to a remote specialist - the specialist reviews the data and provides a diagnostic opinion or treatment recommendations [50]
Remote patient monitoring	- ongoing monitoring of the patient’s condition after treatment - usually delivered through mobile applications or secure online platforms, through which patients send photographs or progress updates [51]
Mobile health (m-health)	- use of mobile digital platforms and applications for healthcare - supports patient education, appointment scheduling, monitoring of oral hygiene and direct communication with the dental team [52]
**Applications**	
Screening and early diagnosis	- early detection and preliminary diagnosis of caries, periodontal disease and other oral lesions from patient-submitted images [47]
Consultations and triage	- remote assessment of symptoms to decide whether urgent in-person examination or treatment is needed [47]
Education and prevention	- education on oral hygiene, nutrition and prevention of oral disease [47]
Orthodontic or postoperative treatment monitoring	- patients send photographs or updates during orthodontic or postoperative care, reducing the number of in-person visits [47]

**Table 3:** Types of intraoral sensors and biomarkers.

**Types of Intraoral Sensors**	**Mechanism**	**Applications**
Chemical sensors	detect chemical components of saliva, such as glucose, lactate, pH and electrolytes	- provide information on oral health and some systemic conditions [58] - detect changes in pH or microbial activity that indicate early caries [59]
Biosensors	biological recognition element (e.g., enzymes, antibodies and DNA) integrated with an electronic transduction mechanism	detection of pathogens or disease-specific biomarkers [60]
Mechanical sensors	measure occlusal forces or mechanical stress on teeth	- used mainly in orthodontics and implant dentistry [61] - help to optimize treatment [62]
Optical sensors	use fluorescence or spectroscopy	detect changes in dental tissues or the presence of biofilm [63]
**Types of biomarkers**	**Examples**	**Applications**
Inflammatory biomarkers	interleukin-1β (IL-1β), interleukin-6 (IL-6) and tumor necrosis factor alpha (TNF-α). [64]	- detection of gingival disease - early diagnosis of periodontitis [65]
Microbiological biomarkers	specific bacteria linked to oral health issues, such as *Porphyromonas gingivalis*, *Treponema denticola*, and *Tannerella forsythia* [66]	- detection of systemic conditions, including diabetes, cancer and viral infections [67] - assessment of biofilm accumulation and personalized feedback on oral hygiene [68]
Genetic biomarkers	gene mutations, single-nucleotide polymorphisms (SNPs) and other genetic variations	identify inherited susceptibility to dental or periodontal disease [69]
Tumor biomarkers	p53, CD44 and salivary microRNAs	early detection of oral cancer [70]

CD44, cluster of differentiation 44; DNA, deoxyribonucleic acid; IL, interleukin; miRNA, microRNA; SNP, single-nucleotide polymorphism; TNF-α, tumor necrosis factor alpha. Most applications listed remain experimental or at the stage of pilot validation; their level of evidence is summarized in Section 7.

**Table 4:** Key studies informing the integration of oral and metabolic data, grouped by type of evidence.

Evidence Category	Study	Design; Population (*n*)	Technology/Data	Principal Finding	Validation/Reference	Main Limitations
Association	Moradpour et al., 2023 [72]	Cross-sectional; Kurdish Dehgolan cohort, adults 35–70 years (3996)	Questionnaire and dental examination; NCEP ATP III	MetS more frequent with less frequent brushing or flossing and with missing teeth	Not applicable	Cross-sectional; self-reported hygiene; single region
Combined prediction model (oral → MetS)	Boitor et al., 2023 [22]	Cross-sectional; hospitalized adults 45–79 years, Romania (296)	AutoML (best: XGBoost) with SHAP; periodontal indices, cardiovascular risk, EQ-5D-5L	Periodontal severity among the most influential predictors of MetS	Internal (70/30 split)	Small hospital sample; 100% test accuracy suggests overfitting; no external validation
Combined prediction model (metabolic → periodontitis)	Wilensky et al., 2023 [73]	Cross-sectional analysis of linked dental–medical records; military personnel, Israel (57,496)	XGBoost feature ranking	Obesity, obstructive sleep apnea and NAFLD most important features	Internal	Young military population; limited generalizability
Combined prediction model (metabolic → periodontitis)	Vu et al., 2025 [74]	Cross-sectional; NHANES 2009–2014, US adults	Several ML classifiers	Periodontal disease status predicted from survey and examination data	Internal	Cross-sectional; no external validation
Combined prediction model (method comparison)	Soysal and Öner, 2026 [76]	Cross-sectional; NHANES (1852)	Regression vs. RF vs. DL; HbA1c and BMI	RF unstable; regression showed consistent rise of periodontal risk with HbA1c	Comparison of models in the same data	Few predictors; cross-sectional
Salivary biomarker (diagnostic/monitoring)	Calixto et al., 2025 [77]	Scoping review of 57 studies; type 1, type 2 and gestational diabetes	Salivary glucose assays	Consistently positive correlation with blood glucose	Various	Wide variation in sensitivity, specificity and methods; not MetS-specific
Salivary biomarker (diagnostic/monitoring)	Shettigar et al., 2024 [78]	Cross-sectional; with and without diabetes (166)	Salivary vs. blood glucose	Weak correlation in people without diabetes; better suited to monitoring	Blood glucose	Correlation only; screening accuracy not established
Salivary biomarker (sensor)	Panwar et al., 2025 [79]	Device validation; with and without diabetes	Optical salivary glucose biosensor	r = 0.97/0.90 with diabetes; 0.58/0.87 without diabetes	Commercial glucometer	Non-laboratory reference; correlation only; not MetS
Integrated screening pathway	None identified	—	—	—	—	Main evidence gap: no prospective study or trial

AutoML, automated machine learning; BMI, body mass index; DL, deep learning; EQ-5D-5L, EuroQol 5-dimension, 5-level questionnaire; HbA1c, glycated hemoglobin; MetS, metabolic syndrome; ML, machine learning; NAFLD, non-alcoholic fatty liver disease; NCEP ATP III, National Cholesterol Education Program Adult Treatment Panel III; NHANES, US National Health and Nutrition Examination Survey; r, correlation coefficient; RF, random forest; SHAP, SHapley Additive exPlanations; XGBoost, extreme gradient boosting.

**Table 5:** Applications of smart technologies in MetS and oral health classified by current level of evidence.

Level of Evidence	Applications	Main Supporting Evidence
Established or routinely used in clinical practice	Measurement of MetS components (waist circumference, blood pressure, glucose and lipids); clinical periodontal examination (probing depth, attachment loss and bleeding on probing); tele-dentistry for consultation and triage	Guidelines and routine care [3]; overviews of systematic reviews of tele-dentistry [53]
Supported by clinical studies but requiring further validation	ML models for MetS prediction (mostly internally validated); wearable, telemonitoring and app-supported lifestyle programs in MetS; AI detection of caries and periodontal bone loss on radiographs; salivary glucose for monitoring people with known diabetes; salivary inflammatory cytokines for periodontitis	Prediction-model studies [11,12,13,15,18,19,21]; trials and pilot studies [28,31,37,38]; diagnostic-accuracy studies and reviews [39,42,43,44,45,46]; correlation studies [77,78,79]; biomarker studies [64,65]
Experimental or proposed future applications	Combined oral–metabolic prediction models; intraoral chemical, optical and microbiome sensors; sweat-based biomarker sensors; salivary multi-marker panels for MetS; integrated dental–medical screening pathways (Figure 3)	Cross-sectional modeling studies [22,73,74,76]; prototype and scoping studies [25,57,71]; no prospective evaluation identified

AI, artificial intelligence; MetS, metabolic syndrome; ML, machine learning. Classification reflects the evidence identified in this review and is intended as a guide, not as a formal grading of certainty.

**Figure A1:**
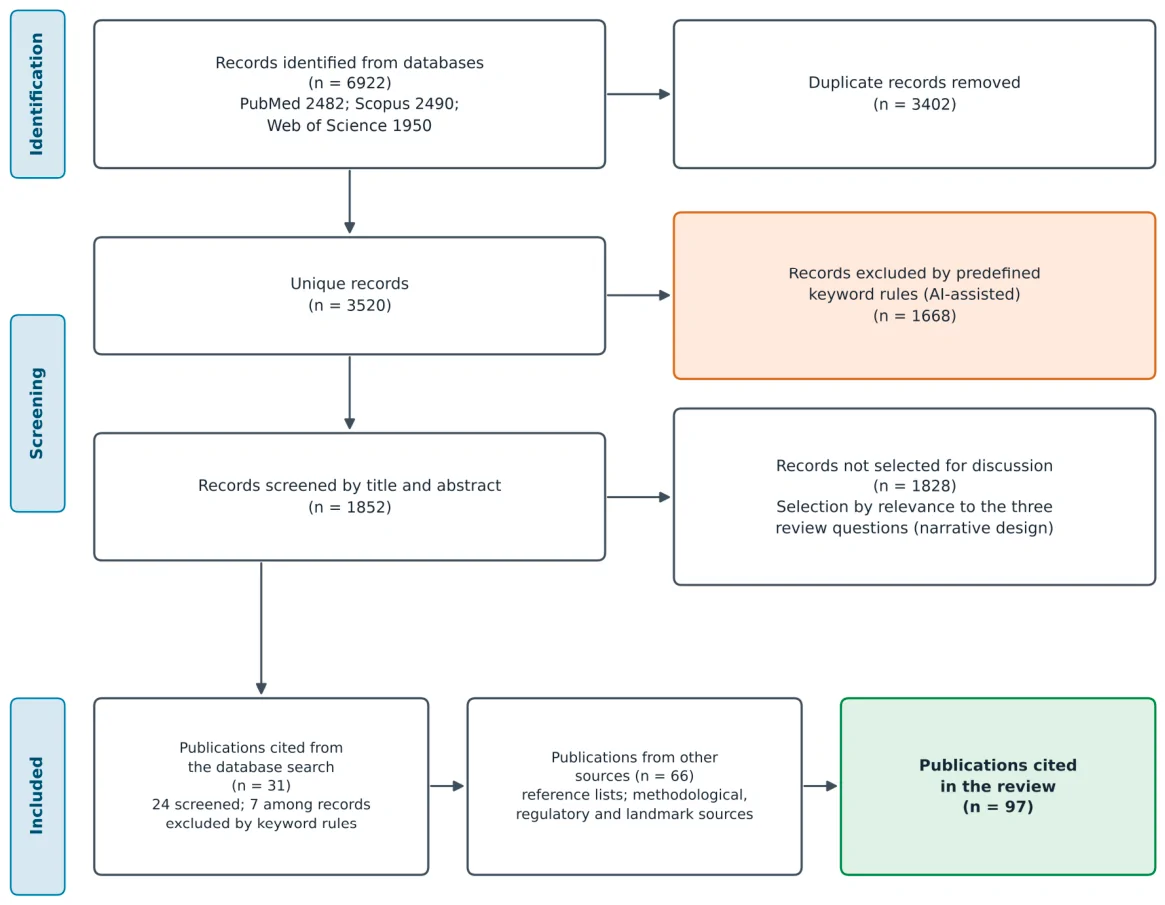
Flow of records through the literature search and selection. AI, artificial intelligence.

## Data Availability

No new data were created or analyzed in this study. Data sharing is not applicable to this article.

## Appendix A

**Table A1:** Search strategies for each database.

Database	Search String	Filters	Date Searched	Records
PubMed	(“Metabolic Syndrome”[MeSH] OR “metabolic syndrome”[tiab] OR “insulin resistance syndrome”[tiab] OR “Oral Health”[MeSH] OR “oral health”[tiab] OR “Periodontitis”[MeSH] OR periodontitis[tiab] OR “periodontal disease*”[tiab] OR “Dentistry”[MeSH] OR dentistry[tiab] OR “Dental Caries”[MeSH] OR “dental caries”[tiab] OR “oral disease*”[tiab]) AND (“artificial intelligence”[ti] OR “machine learning”[ti] OR “deep learning”[ti] OR wearable*[ti] OR “internet of things”[ti] OR telemedicine[ti] OR telehealth[ti] OR “mobile health”[ti] OR mhealth[ti] OR “mobile app*”[ti] OR “smartphone app*”[ti] OR teledentistry[ti] OR “tele-dentistry”[ti] OR biosensor*[ti] OR “intraoral sensor*”[ti] OR “remote monitoring”[ti]) AND (“2021/01/01”[dp] : “2026/05/31”[dp]) AND English[lang] NOT (editorial[pt] OR letter[pt] OR comment[pt])	Included in the string: publication date, English, exclusion of editorials, letters and comments	26 September 2026	2482
Scopus	TITLE-ABS-KEY(“metabolic syndrome” OR “insulin resistance syndrome” OR “oral health” OR periodontitis OR “periodontal disease*” OR dentistry OR “dental caries” OR “oral disease*”) AND TITLE(“artificial intelligence” OR “machine learning” OR “deep learning” OR wearable* OR “internet of things” OR telemedicine OR telehealth OR “mobile health” OR mhealth OR “mobile app*” OR “smartphone app*” OR teledentistry OR “tele-dentistry” OR biosensor* OR “intraoral sensor*” OR “remote monitoring”) AND PUBYEAR > 2020 AND PUBYEAR < 2027 AND LANGUAGE(English) AND (DOCTYPE(ar) OR DOCTYPE(re))	Included in the string: publication year 2021–2026, English, articles and reviews; records after 31 May 2026 removed at screening	26 September 2026	2490
Web of Science Core Collection	TS=(“metabolic syndrome” OR “insulin resistance syndrome” OR “oral health” OR periodontitis OR “periodontal disease*” OR dentistry OR “dental caries” OR “oral disease*”) AND TI=(“artificial intelligence” OR “machine learning” OR “deep learning” OR wearable* OR “internet of things” OR telemedicine OR telehealth OR “mobile health” OR mHealth OR “mobile app*” OR “smartphone app*” OR teledentistry OR “tele-dentistry” OR biosensor* OR “intraoral sensor*” OR “remote monitoring”) AND DOP=(2021-01-01/2026-05-31) AND LA=(English) AND DT=(Article OR Review)	Included in the string: publication date, English, articles and reviews	26 September 2026	1950
Total	—	—	—	6922 (3520 after removal of 3402 duplicates)

MeSH, Medical Subject Headings; dp, date of publication; lang, language; pt, publication type; ti/TI/TITLE, title field; tiab, title/abstract field; TITLE-ABS-KEY, title, abstract and keywords; TS, topic field. Truncation (*) retrieves all word endings.

**Table A2:** Main risk-of-bias signals in the prediction-model studies of Table 1 (qualitative appraisal informed by PROBAST).

Study	Outcome	Defining MetS Components Among Predictors	Validation	Main Concerns (Analysis Domain)	Overall Concern
Pawade et al., 2024 [15]	Prevalent	Yes (waist circumference)	Internal (hold-out)	Training–test accuracy gap (95% vs. 88.97%); data source not reported; accuracy as main metric	High
Akbarzadeh et al., 2022 [11]	Prevalent	Likely (clinical variables)	Internal (repeated CV)	No external validation; several algorithms compared in the same data	Unclear to high
Li et al., 2024 [12]	Prevalent	Possible (LASSO selection from 59 variables)	External (three cohorts)	Best algorithm varied between cohorts; populations and definitions differ	Lower
Mirzaei et al., 2025 [18]	Prevalent	Yes (waist, glucose, triglycerides and blood pressure)	Not reported	Circular prediction of a definition-based outcome	High
Tavares et al., 2022 [19]	Incident	Likely (clinical and laboratory variables)	Internal	Repeated visit pairs per individual (possible leakage if split by visit); external validation recommended by the authors	Unclear
Shin D, 2024 [13]	Incident	No (demographic, lifestyle, dietary and polygenic score)	Internal	Near-perfect AUC (0.994) with many genetic predictors	High
Shin H et al., 2023 [21]	Prevalent	Yes (waist circumference and blood pressure)	Internal (10% hold-out)	Partial circularity; large sample reduces overfitting risk	Unclear
Boitor et al., 2023 [22]	Prevalent	Partly (cardiovascular risk)	Internal (70/30 split)	100% test accuracy in about 89 test patients; AutoML model selection	High

AUC, area under the receiver operating characteristic curve; AutoML, automated machine learning; CV, cross-validation; LASSO, least absolute shrinkage and selection operator; PROBAST, Prediction model Risk Of Bias ASsessment Tool. Judgements are qualitative and based on the information summarized in Table 1; a full PROBAST assessment would require item-level extraction from each full text. High = at least one major signal (very high performance with internal validation only, circular prediction of prevalent MetS or a very small test set); lower = external validation in independent cohorts.
